# Lack of consensus in inter-laboratory haematology results in selected laboratories in the southern and northern zones of Ghana

**DOI:** 10.4314/gmj.v57i3.8

**Published:** 2023-09

**Authors:** Ibrahim B Halidu, Amos X Gafa, Samuel D K Blanney, Benjamin T Barimah, David Akan-Enge, Joseph Boachie, Kate A Kontor, Patrick Adu

**Affiliations:** 1 Department of Medical Laboratory Science, School of Allied Health Sciences, University of Cape Coast, Ghana; 2 Department of Biomedical Sciences, School of Allied Health Sciences, University of Cape Coast, Ghana; 3 Medical OPD, Cape Coast Teaching Hospital, Cape Coast, Ghana

**Keywords:** intra-laboratory reproducibility, laboratory harmonisation, External quality assessment scheme

## Abstract

**Objective:**

To assess the inter-laboratory comparability and intra-assay reproducibility of full blood count (FBC) results.

**Design:**

Exploratory cross-sectional study.

**Setting:**

Three and two selected medical laboratories in the northern and southern zones, respectively.

**Participants:**

Forty-nine individuals per zone; 16 type 2 diabetes mellitus, 16 with HbAS haemoglobin type and 17 normal samples

**Intervention:**

Each sample was run eleven times through the analysers in the participating laboratories to evaluate intra-laboratory reproducibility and comparability of FBC results.

**Main Outcome Measure:**

Intra-laboratory reproducibility was evaluated using %coefficient variation (%CV). Interlaboratory comparisons were assessed through t-test or One-Way ANOVA for two-sample and three-sample tests. All statistical testing was undertaken using the two-tailed assumption.

**Result:**

Statistically significantly different haemoglobin levels were estimated in both northern and southern zones (mean difference 0.00 g/dL to 3.75 g/dL vs 0.18 g/dL to 1.92 g/dL respectively). Also, total WBC counts significantly differed across laboratories in both northern and southern zones (mean difference 0.15 ×10^9^/L - 3.86 ×10^9^/L vs 0.02 ×10^9^/L to 1.39 ×10^9^/L respectively). Furthermore, platelet counts significantly differed across the participating laboratories in the northern and southern zones (mean difference 0.40 ×10^9^/L to 299.76 ×10^9^/L vs 5.7 ×10^9^/L to 76.9 ×10^9^/L respectively). Moreover, there was evidence of non-reproducibility of results within the respective laboratories in each zone as the respective %CV were outside the acceptable limits.

**Conclusion:**

The intra-laboratory non-reproducibility and inter-laboratory non-comparability of FBC results highlight the need to establish a national quality assessment scheme to harmonise laboratory practices nationwide.

**Funding:**

This study was funded by the University of Cape Coast Individual-Led Research Support Grant (RSG-INDI-CoHAS-2019-107).

## Introduction

Globally, medical laboratories play a central role in evidence-based medicine. Substantive diagnoses are generally made based on the evidence of credible and timely laboratory results emanating from the processing of patients' samples.^1^ One key component of the medical laboratory is the haematology laboratory which draws patient blood and investigates for evidence of disease to aid diagnosis. Generally, each laboratory should use quality assurance principles and standard operating procedures to regulate all aspects of laboratory services, including training and staff recruitment, equipment procurement and maintenance, daily operation of laboratory services, and reporting of laboratory results. Faithful adherence to these principles ensures inter-laboratory harmonisation, which practically translates that the sample analysed in different laboratories within an acceptable timeframe will yield reproducible and comparable results. Standardization in laboratory practice is advocated and mandated by accredited international authorities (e.g. the WHO, the International Organisation for Standardisation, and the European Committee on Harmonization) to reduce/prevent clinical laboratory errors.^2^

Errors in the clinical haematology laboratory reports could lead to significant adverse effects on patients as it might result in misdiagnosis, inappropriate medical interventions as well as inappropriate use of funds by both government and patients. 3, 4 To maintain adherence to these high standards, laboratories are mandated to subscribe to external quality assurance assessment schemes as a means of assuring continuous competence of laboratory staff, validity and reliability of results as well as comparability of inter-laboratory results.^5^ Even in developed countries where this directive is strictly adhered to, it is recognized that only an objective external quality assurance (EQA) scheme could detect deviation from standard practices.^6^

For example, the international society of laboratory haematology (ISLH) recommends a subscription to the annual EQA program by laboratories. Even where this is not practicable because of logistical challenges, there are additional recommendations that laboratories should compare their performance with other laboratories to ensure the reliability of their findings.^7^ More importantly, participation in EQA schemes is mandatory for laboratory accreditation or certification as clearly stated in the International Organisation for Standardisation (ISO) 9000, ISO Guides 25 and 58, as well as ISO 15189.^8^ However, in sub-Saharan Africa, since authorised/centralised agencies that continuously assess the operation of haematology laboratories are non-existent, there is no formalised means of assessing outputs from these laboratories to ensure inter-laboratory harmonisation. Literature on studies that assessed the operations of various haematology laboratories in Ghana is non-existent. The only study that has attempted such a quality assessment study among haematology laboratories in Ghana used fixed blood cells when the country was still largely using manual methods.^9^ Since most of the haematology laboratories in the country are now using automated analysers, there is an urgent need to address this issue of comparability and inter-laboratory harmonisation. Besides, that Opoku-Okrah et al 9 study also used fixed samples which does not reflect the rheological properties of unfixed samples that are routinely handled by these laboratories.^2^,^5^ In our view, this study is the first to systematically investigate the operations of automated analysers used within haematology laboratories in the Southern and Northern zones of Ghana and thus begin the process of addressing the issue of intra-laboratory reproducibility and inter-laboratory comparability of results as well as harmonisation of laboratory practice.

## Methods

### Study Design

This exploratory cross-sectional study investigated the intra-assay reproducibility and the comparability of haematology results between laboratories involved. Overall, five (5) laboratories were sampled; three in the Northern and two in the Southern zones of Ghana.

All five laboratories were each using a 3-part haematology analyser. In order to ensure the validity of inter-laboratory comparison, samples were run in the participating laboratories within 2 - 4 hours. Therefore, laboratories in each zone were compared to respective laboratories in the same zone. The sampling period began in November 2020 and ended in April 2021.

### Study Area

The Central and the Bono East regions were conveniently selected to represent the southern and northern zones, respectively. Introductory letters were sent to the heads of the laboratories through the regional laboratory associations; only laboratories that consented to be part of this inter-laboratory comparative study were eligible. However, to prevent storage-induced haematological changes, for laboratories to be included in the study, they should be close in proximity to ensure that split samples could reach the laboratories within an hour interval for onward processing.

### Participating laboratories

Three medical laboratories that participated in the Bono East for this exploratory study are herein identified as KMH-L (a government facility laboratory), DET-L (a private laboratory) and AMH-L (a faith-based facility laboratory), representing all the various ownerships of Ghana healthcare delivery. The two medical laboratories in the Cape Coast Metropolis that participated in the study are herein identified as EPC-L (a government facility laboratory) and BGH-L (a private hospital laboratory). In each zone, the participating laboratories were close to allowing sample processing within the stipulated 2 - 4 hours. The two laboratories within the Cape Coast metropolis were about 4.8 km apart and required approximately 10 minutes of drive time. Two of the three laboratories in the Bono East region were in Kintampo and required 15 minutes' drive time. In contrast, the other laboratory was in Techiman (about 70.4 km) and required approximately 1 hour drive time.

The participating laboratories and the haematology analysers used were DET-L (BC-10; Mindray, China), AMH-L (BC-3000 Plus; Mindray, China), EPC-L (BC-2800; Mindray, China), KMH-L and BGH-L (3000 Plus; Urit, China).

### Sampling Procedure

The International Committee for Standardisation in Haematology (ICSH)^10^ recommends that inter-laboratory verification studies should include samples from normal populations and pathologic samples (in the study, diabetes mellitus) to represent the population that the laboratories serve. Additionally, since most haematology analysers operate on the principle of sphering, samples from sickle cell trait patients were included to assess inter-laboratory performance in individuals with red cells that may be largely resistant to sphering.

In each zone, 49 participants were recruited: 17 with no clinically diagnosed metabolic disease, 16 with clinically diagnosed type 2 diabetes and 16 with Hb AS haemoglobin type. Each sample was run 11X through the analysers in the participating laboratories to evaluate reproducibility (intra-laboratory) and the comparability of inter-laboratory performances at low and high analytical ranges.

### Blood Sample Collection, Preparation and Processing

Each participant had four (4) ml of venous blood drawn into an ethylenediamine tetraacetic acid (EDTA) anticoagulated tube and run on the haematology analysers within the same zone within two hours, in accordance with standard protocols. This exploratory study recruited 33 participants (17 normal samples and 16 samples from type 2 diabetes mellitus patients) in each zone. Additionally, since most haematology analysers operate on the principle of sphering, 16 samples from sickle cell trait patients were included to assess inter-laboratory performance in individuals with red cells that may be largely resistant to sphering. In order to ensure that the samples selected adequately represented the various concentrations assayed by the participating laboratories, daily samples with low, normal and high blood count parameters were assayed to evaluate their performances at low and high analytical ranges. Specifically for high haemoglobin levels, a specimen with normal haemoglobin was centrifuged, and about 400 µl of plasma was pipetted out. Afterwards, the sample was thoroughly re-mixed and then run to provide a surrogate sample to mimic how the analyser would handle such a high haemoglobin level regarding reproducibility. For high total WBC counts, the sample was purposely selected from routine patient samples with counts higher than the upper limit of the reference range.

### Haemoglobin electrophoresis

The haemoglobin type of participants was determined using the cellulose acetate electrophoresis procedure as previously described.^6^ For each electrophoretic run, control samples composed of haemoglobin A, C, S, and F were run to enable haemoglobin identification.

As an additional quality control procedure, all samples that typed as haemoglobin AS were additionally screened using the sickling slide test to confirm that the haemoglobin variant determined was indeed haemoglobin S. This helped rule out the possibility of other haemoglobins that co-migrate with haemoglobin S during electrophoresis at basic pH.

### Ethical Clearance

The protocols for the study were approved by the Institutional Review Board of the University of Cape Coast (UCCIRB/EXT/2019/32). Permission was again sought from the respective Management of the hospitals and Laboratory Managers before the start of the study. Written informed consent was sought from participants after a thorough explanation of the study. Participants agreed by written consent to partake in the study. The anonymity of participants was preserved.

### Quality assurance

All samples were transported in portable ice chests containing ice packs to ensure that samples were maintained at 4 – 6°C temperature. All samples were analysed within 2 – 4 hours of venesection to prevent storage-related changes. Participant selection covered healthy individuals, those with metabolic diseases (diabetes), and inherited genetic disorders to replicate the spectrum of specimens that these laboratories handle daily. Since it is extensively reported in the literature that different well-calibrated haematology analysers handle preserved blood differently, 6, 7 it was ensured that this study only used freshly drawn blood samples to prevent such confounding machine-derived variability. Before running samples, the samples were thoroughly mixed and allowed to equilibrate to room temperature to mimic how samples are normally processed in the laboratory. This was undertaken to prevent storage-related artefacts that can cause variability in the analysis process and confound the interpretation of results.

### Data analysis

The data obtained from each haematology analyser was entered into Microsoft Excel, checked for completeness, and analysed using GraphPad Prism version 8 for Windows (GraphPad Inc., USA). The normality of the data obtained was tested using the D'Agostino & Pearson omnibus normality test. To estimate the intra-laboratory reproducibility of results issued by each participating laboratory, the coefficient of variation (CV%) was calculated for each sample using the eleven (11) replicates run per analyser. The %CV was calculated as CV = (SD/mean) * 100%, where SD = standard deviation. The calculated CV% was compared with the referenced CV% as published previously.^6^

Specifically, the acceptable CV% are 3 – 4% (haemoglobin concentration and RBC counts), 4 – 5 % ((PCV, MCH, MCH, MCHC), 8 – 10% (total leucocyte count) and 10 – 15% (platelet count).

The comparability of inter-laboratory performances was evaluated using the One-Way ANOVA (with Tukey correction for multiple comparisons) or the unpaired T-test for three-sample and two-sample comparisons, respectively. All statistical significance was established at p< 0.05 at a 95% confidence interval using the two-tailed assumptions.

## Results

The mean haemoglobin levels estimated per 11 replicate runs per the analyser being used in the respective laboratories are compared in Table 1. Significant statistical differences in the mean haemoglobin values were estimated for the same samples in the participating laboratories in both zones. In the three participating laboratories in the northern zone, the mean haemoglobin levels were estimated with a mean difference of between 0.00 g/dL to 3.75 g/dL compared to 0.18 g/dL to 2.36 g/dL mean difference across the two participating laboratories in the southern zone. Additionally, the estimated coefficient of variations for two samples with high haemoglobin (#1136) and low haemoglobin (#932) were above the acceptable limits in laboratories DET-L and KMH-L, suggesting non-reproducibility of haemoglobin values from the respective analysers. In the two participating laboratories in the southern zone, three samples #003 and #012 (for laboratory EPC-L) and #001 (for BGH-L) had coefficients of variation above the acceptable limits of 3 – 4%.

**Table 1:** Intra-and inter-laboratory comparison of haemoglobin results

	DET-L (g/dL)	KMH-L (g/dL)	AMH-L (g/dL)	Mean difference range (g/dL)	P1	P2	P3
Northern zone							
**#1136**	20.91 **(10.78)**	19.99 **(4.91)**	17.16 (0.60)	0.92 – 3.75	ns	<0.0001	0.0002
**#1114**	13.78 (1.12)	13.32 (1.20)	12.70 (2.28)	0.46 – 1.08	<0.0001	<0.0001	<0.0001
**#1117**	8.09 (1.03)	8.87 (3.07)	9.30 (2.54)	0.78 – 1.21	<0.0001	<0.0001	0.0002
**#1738**	11.45 (1.43)	11.62 (2.63)	11.19 (1.02)	0.17 – 0.43	ns	0.0217	0.0001
**#1730**	9.56 (2.11)	8.92 (1.86)	9.15 (1.79)	0.41 – 0.64	<0.0001	<0.0001	0.0146
**#932**	3.46 **(6.74)**	3.87 **(4.49)**	3.62 (1.67)	0.25 – 0.41	<0.0001	ns	0.0043
**#549**	7.54 (1.07)	7.90 (1.39)	7.96 (2.47)	0.06 – 0.42	<0.0001	<0.0001	ns
**#526**	11.83 (1.26)	11.85 (2.91)	11.83 (1.30)	0.00 – 0.02	ns	ns	ns
**#616**	12.26 (1.33)	12.11 (1.40)	12.60 (0.79)	0.15 – 0.34	ns	<0.0001	<0.0001
**#1679**	10.04 (1.02)	10.31 (2.31)	9.68 (2.00)	0.27 – 0.63	0.0051	0.0003	<0.0001
**Southern zone**							
	**EPC-L**	**BGH-L**					
**#001**	11.25 (2.19)	10.33 **(17.80)**		0.92	ns		
**#002**	12.75 (2.23)	10.84 (1.90)		1.91	<0.0001		
**#003**	13.18 **(4.08)**	11.26 (1.45)		1.92	<0.0001		
**#004**	10.16 (2.26)	8.86 (1.06)		1.30	<0.0001		
**#005**	11.23 (1.13)	10.32 (1.55)		0.91	<0.0001		
**#007**	11.95 (1.26)	10.91 (1.12)		1.04	<0.0001		
**#012**	12.61 **(8.05)**	10.25 (1.10)		2.36	<0.0001		
**#017**	13.35 (1.52)	12.63 (1.59)		0.72	<0.0001		
**#018**	13.07 (3.54)	12.35 (1.37)		0.72	<0.0001		
**#019**	13.26 (5.24)	13.44 (4.56)		0.18	ns		

Data is presented as mean (%CV); %CV was calculated as CV = (SD/mean) * 100%. SD = standard deviation. Three sample comparisons were undertaken using One-Way ANOVA with Tukey's correction for multiple comparisons; all two-sample comparisons were undertaken using the unpaired T-test; All statistical comparisons were undertaken using the two-tail assumptions.

Table 2 shows the mean white blood cell count estimated per 11 replicate runs per the respective laboratories' analysis. The total white blood cell counts also demonstrated statistically significant differences across the respective laboratories. Overall, the total white blood cell counts were estimated across the three participating laboratories in the northern zone with a mean difference of between 0.15 ×10^9^/L to 3.86 ×10^9^/L compared to the two participating laboratories in the southern zone where the mean difference was between 0.02 ×10^9^/L to 1.39 ×10^9^/L. Moreover, when the intra-laboratory reproducibility of total white blood cell counts using the coefficient of variations, whereas only one laboratory (DET-L) in the northern zone demonstrated non-reproducibility for two samples (#533 and #006), both laboratories in the southern zone (EPC-L: #012; BGH-L: #012 and #018) demonstrated non-reproducibility of white blood cell counts.

**Table 2:** Intra-and inter-laboratory comparison of total white blood cell count

	DET-L (×10^9^/L)	KMH-L (×10^9^/L)	AMH-L (×10^9^/L)	Mean difference range (×10^9^/L)	P1	P2	P3
**Northern zone**							
**#533**	18.71 **(12.27)**	21.21 (0.71)	19.11 (7.40)	**2.10 – 2.50**	**0.0021**	ns	**0.0098**
**#722**	2.32 (3.24)	2.54 (4.42)	2.39 (2.93)	**0.15 – 0.22**	**<0.0001**	ns	**0.0015**
**#006**	7.32 **(29.85)**	9.12 (1.37)	8.30 (2.35)	**0.82 – 1.80**	**0.0064**	ns	ns
**#007**	2.33 (8.17)	4.79 (1.97)	5.67 (2.10)	**0.88 – 3.34**	**<0.0001**	**<0.0001**	**<0.0001**
**#010**	3.30 (3.03)	3.56 (2.59)	4.64 (4.55)	**1.08 – 1.34**	**0.0005**	**<0.0001**	**<0.0001**
**#013**	3.30 (5.91)	3.55 (2.31)	4.76 (3.57)	**1.21 – 1.46**	**0.0026**	**<0.0001**	**<0.0001**
**#003**	2.68 (4.66)	5.01 (2.75)	6.54 (2.67)	**1.53 – 3.86**	**<0.0001**	**<0.0001**	**<0.0001**
**#2319**	3.66 (2.52)	4.20 (2.82)	4.56 (2.84)	**0.36 – 0.90**	**<0.0001**	**<0.0001**	**<0.0001**
**#001**	3.90 (2.29)	4.16 (1.26)	3.60 (3.03)	**0.30 – 0.56**	**<0.0001**	**<0.0001**	**<0.0001**
**#616**	4.02 (2.91)	5.15 (1.34)	3.37 (3.41)	**0.65 – 1.78**	**<0.0001**	**<0.0001**	**<0.0001**
**Southern zone**							
	**EPC-L**	**BGH-L**					
**#001**	8.06 (1.79)	6.67 (4.03)		**1.39**	**<0.0001**		
**#002**	3.47 (5.48)	3.51 (3.71)		0.04	ns		
**#003**	6.31 (8.87)	5.70 (1.57)		**0.61**	**0.0019**		
**#004**	5.28 (4.85)	4.79 (2.55)		**0.49**	**<0.0001**		
**#005**	4.75 (1.97)	4.62 (3.36)		**0.13**	**0.043**		
**#007**	5.60 (2.65)	5.72 (2.04)		0.12	ns		
**#012**	6.40 **(14.71)**	6.53 **(13.30)**		0.13	ns		
**#017**	5.61 (7.84)	5.46 (8.24)		0.15	ns		
**#018**	8.56 (2.36)	7.78 **(12.19)**		**0.78**	**0.0156**		
**#019**	6.02 (3.14)	6.01 (3.02)		0.01	ns		

Data is presented as mean (%CV); %CV was calculated as CV = (SD/mean) * 100%. SD = standard deviation. Three sample comparisons were undertaken using One-Way ANOVA with Tukey's correction for multiple comparison; all two-sample comparisons were undertaken using the unpaired T-test; All statistical comparisons were undertaken using the two-tail assumptions.

Table 3 shows the mean platelet count estimated per the 11 replicate runs per the analyser being used in the respective laboratories. In the participating laboratories in the Northern zone, the platelet counts demonstrated evidence of non-reproducibility of intra-laboratory results as the CV% exceeded acceptable limits.

**Table 3:** Intra-and inter-laboratory comparison of platelet counts

	DET-L (×10^9^/L)	KMH-L (×10^9^/L)	AMH-L (×10^9^/L)	Mean difference range (×10^9^/L)	P1	P2	P3
**#533**	90.45 **(21.24)**	74.00 **(17.47)**	88.45 **(20.33)**	2.00 – 16.45	ns	ns	ns
**#722**	80.36 **(23.78)**	94.09 **(19.78)**	152.3 (9.69)	13.73 – 71.94	ns	**<0.0001**	**<0.0001**
**#006**	75.64 **(42.92)**	255.0 (10.86)	375.4 (4.70)	**120.4 – 299.76**	**<0.0001**	**<0.0001**	**<0.0001**
**#007**	142.5 **(27.71)**	262.90 (13.13)	211.00 (3.99)	**68.50 – 120.40**	**<0.0001**	**<0.0001**	**0.0012**
**#010**	197.90 (3.35)	247.20 (3.55)	293.50 (10.04)	**46.30 – 95.60**	**<0.0001**	**<0.0001**	**<0.0001**
**#013**	213.6 (8.08)	140.00 **(25.60)**	259.40 (5.74)	**45.80 – 119.4**	**<0.0001**	**0.0004**	**<0.0001**
**#003**	64.91 **(49.17)**	207.50 (2.43)	145.30 (3.30)	**80.39 – 142.59**	**<0.0001**	**<0.0001**	**<0.0001**
**#2319**	111.10 (9.83)	135.60 (4.30)	146.50 **(24.26)**	**10.90 – 35.40**	**0.0332**	**0.0017**	ns
**#001**	208.50 (3.29)	279.60 (2.64)	227.60 (2.72)	**19.10 – 71.10**	**<0.0001**	**<0.0001**	**<0.0001**
**#616**	285.90 (3.41)	286.30 (3.69)	309.80 (3.74)	0.40 – 23.90	ns	**<0.0001**	**<0.0001**
	**EPC-L**	**BGH-L**					
**#001**	383.2 (1.40)	450.3 (1.24)		**67.10**	**<0.0001**		
**#002**	183.2 (2.11)	193.0 (1.09)		**9.80**	**<0.0001**		
**#003**	205.5 (3.65)	241.1 (2.53)		**35.60**	**<0.0001**		
**#004**	81.2 (3.43)	93.4 (3.47)		**12.20**	**<0.0001**		
**#005**	335.1 (0.97)	412.0 (1.49)		**76.90**	**<0.0001**		
**#007**	420.7 (2.95)	457.9 (3.46)		**37.20**	**<0.0001**		
**#012**	180.7 (6.79)	239.3 (11.62)		**58.60**	**<0.0001**		
**#017**	252.6 (3.36)	261.0 (2.45)		**8.40**	**0.0168**		
**#018**	180.5 (4.40)	189.3 (5.07)		**8.80**	**0.0309**		
**#019**	229.0 (8.68)	213.3 (5.05)		**5.70**	**0.0246**		

Data is presented as mean (%CV); %CV was calculated as CV = (SD/mean) * 100%. SD = standard deviation. Three sample comparisons were undertaken using One-Way ANOVA with Tukey's correction for multiple comparison; all two-sample comparisons were undertaken using the unpaired T-test; All statistical comparisons were undertaken using the two-tail assumptions.

However, this was not the case in the two participating laboratories in the southern zone where the estimated CV% were within range. Overall, the platelet counts were estimated across the three participating laboratories in the northern zone with a mean difference of between 0.40 ×10^9^/L (lowest) to 299.76 ×10^9^/L (highest) compared to the two participating laboratories in which the mean difference was between 5.7 ×10^9^/L to 76.9 ×10^9^/L.

## Discussion

Adherence to quality assurance and quality control principles in the haematology laboratory are major steps towards inter-laboratory harmonisation in which the same sample analysed in different laboratories will yield reproducible and comparable results. Irrespective of the strict implementation and adherence to quality control/assurance procedures across medical laboratories, external quality assessment provides one of the most credible means of identifying laboratories that do not consistently achieve accurate results and, therefore, prevention of systematic bias in generating patient results.^11^,^12^ In sub-Saharan Africa, external quality assessment schemes are not commonplace, given the logistical and legislative challenges. This study sought to investigate the intra-laboratory reproducibility of haematology results and the comparability between laboratories in selected hospitals across Ghana's northern and southern zones. In this study, we used coefficient variation 6 to assess the reproducibility of haematological results within the individual laboratories and the statistical differences in the means of the respective haematological parameters to estimate the inter-laboratory comparison of results. Our results demonstrate the lack of harmonisation of haematology laboratory results intra-laboratory and across the laboratories that participated in the study.

Regarding the reproducibility of laboratory results within a given laboratory, platelet counts appeared to be the most non-reproducible parameter among the haematological parameters on the complete blood count variables. This is evidenced by the high intra-laboratory platelet count coefficient of variation estimated among laboratories in the northern zone above the acceptable intra-laboratory platelet count coefficient of variation (10 – 15%). Practically, this translates into the fact that platelet count estimated from the same patient's sample at seconds intervals will significantly vary from one another. Such intra-laboratory variability indicates systematic errors emanating from the respective haematology analyser used by the individual laboratories. This will affect patient healthcare and warrants urgent attention to ensure that the complete blood count reports always represent the patients' health status. Another piece of evidence that makes the findings graver is the lack of consensus on the inter-laboratory platelet count across the laboratories which was observed in participating laboratories from both southern and northern zones.

This shows that there is a lack of agreement among the laboratories, which raises the question as to which of the laboratories' reports should be taken as representing the true state of the patient in question.

In practical terms, the platelet counts are being estimated across the laboratories with a difference of between 2.00 ×10^9^/L − 16.45 ×10^9^/L. The platelet count for the same specimen was estimated across the three laboratories with a difference of 62.2 ×10^9^/L − 142.30 ×10^9^/L. Implicitly, a specimen would be determined to have a normal platelet count in one laboratory, and that same specimen will be determined to be thrombocytopenic in another laboratory. These significant quantitative differences were also observed among the two laboratories in the southern zone that participated, indicating a systemic problem within and across the laboratories; for example, in one sample, the platelet count was estimated across the laboratories with a difference of 67.1 ×10^9^/L (450.3 − 383.2 ×10^9^/L). The crucial question then is which of the laboratories should be taken to represent the patient's true haematological state since the disparate results could not all accurately represent the patient. These significant quantitative differences cannot be overlooked or trivialised since they could greatly impact patient care decisions and must be urgently addressed by implementing nationally or regionally mandated EQA schemes.

It should be noted that the results presented herein are intra-sample specific %CV calculated per the 11 replicates run on each sample. Since these %CVs are not pooled %CV, which would have been averages of all samples processed, the element of individual sample heterogeneity and hence outliers impacting results interpretation is not a variable to be considered. This is why we are strongly arguing for the country's need to urgently consider implementing EQA to streamline the operations of medical laboratories in Ghana.

The intra-laboratory non-reproducible and disparate inter-laboratory haematology report affected haemoglobin estimation as well. For example, for one specimen, the mean haemoglobin concentration was estimated to be 20.19 g/dl, 19.99 g/dl and 17.16 g/dl, respectively, by DET-L, KMH-L and AMH-L. This translates that the haemoglobin levels were estimated with a difference of 0.92 g/dl to 3.75 g/dl across the laboratories. In another specimen, the mean haemoglobin values estimated across the three laboratories significantly differed (8.09 g/dl vs 8.87 g/dl vs 9.30 g/dl), resulting in a difference of 0.78 g/dl to 1.21 g/L.

A similar trend was repeated across the two laboratories in the southern zone that participated in the study, with haemoglobin estimated with a difference that ranged from 0.72 g/dL to as high as 2.36 g/dL.

These are not small differences considering that the transfusion of one unit of whole blood only increases the haemoglobin concentration by 1 g/dl 13, 14, increment/reduction of a patient's haemoglobin concentration by >1 g/dL should warrant attention. Since the same specimen was run in the respective laboratories following standard preanalytical quality control procedures on the sample handling and processing, these differences could be attributed to analyser-specific issues such as faulty calibration and lack of routine analyser maintenance. Moreover, in addition to the significant statistical differences in the mean inter-laboratory haemoglobin concentrations, there was evidence of non-reproducibility of haemoglobin concentration estimated per sample in the same laboratory. We make this claim based on the intra-laboratory coefficient variation that exceeded the acceptable limits of 3 – 4% and reached 10.78% and 17.80% for some samples run in the northern and southern zones, respectively. Thus, our study has provided evidence that haemoglobin concentrations are estimated non-reproducibly even within the same laboratory and across laboratories in both the participating laboratories in the southern and northern zones. It must be emphasised that haemoglobin estimation is an important consideration in the clinical setting as it serves as a key determinant as to whether to transfuse an anaemic client or not 15; thus, any uncertainties in its determination warrant attention. A previous study in Kenya also found a high %CV (as high as 41% in some cases) in the haemoglobin estimation across 292 laboratories 16, suggesting that our findings further support the need to implement EQA schemes across the sub-region. Although our sample size of two and three laboratories in the southern and northern zones, respectively, precludes us from making far-reaching statements, we are of the view that these disparate results are not isolated cases and might represent a microcosm of an entrenched problem within the medical laboratories operating within the country. We make this supposition based on the fact that no authoritative agency is mandated to enforce external quality assessment schemes and proficiency testing for laboratories across the country that would have identified out-of-consensus laboratories and subsequently design programmes to address these. Without such an agency enforcing the dictates of ISO15789 requirements, each laboratory is left alone, potentially detrimental to patient care and the healthcare system.

Moreover, the non-reproducibility of the variables on the complete count report was also visible for total WBC counts in the participating laboratories from both the southern and northern zones since the coefficient of variations exceeded the acceptable limits of 8 – 10% in some cases. Additionally, there were statistically significant differences in the mean total WBC estimated across the laboratories for the same samples. For example, in the three participating laboratories in the northern zones, total WBC counts were estimated at a mean difference ranging from 0.15 ×10^9^/L to 3.86 ×10^9^/L compared to the 0.01 ×10^9^/L to 1.39 ×10^9^/L difference range determined for the two laboratories in the southern zone. These significant differences in haemoglobin levels, platelet counts, and WBC counts indicate a general lack of consensus in the complete blood count variables reported by the participating laboratories.

The findings presented herein agree with a previous study undertaken in Nigeria that also found statistically significant differences in total white blood counts estimated among three participating laboratories. 17 The greater question that remains to be determined is which of the laboratories could be taken to issue results that are in tune with the patient's physiological state.

Due to logistical and financial challenges, we could not extend the study to cover the entire country, which would have allowed us to decipher the exact problem of lack of harmonisation among medical laboratories nationwide. It must be emphasised that our sample size was further affected by the reluctance of most laboratories to participate in this exploratory study. Additionally, we could not evaluate the accuracy of measurements by using reference materials sent to the participating laboratories. 18 This would have provided the only avenue to establish whether a specific laboratory or all the laboratories were out-of-consensus.

## Conclusion

Despite the limitations discussed above, this study demonstrates the need for the country to establish an external quality assessment scheme and a proficiency testing program to provide oversight responsibilities for medical laboratories. This would go a long way to ensure that laboratory results from these laboratories reflect the state of a patient's health.
